# Agriculture in West Africa in the Twenty-First Century: Climate Change and Impacts Scenarios, and Potential for Adaptation

**DOI:** 10.3389/fpls.2016.01262

**Published:** 2016-08-30

**Authors:** Benjamin Sultan, Marco Gaetani

**Affiliations:** ^1^Sorbonne Universités, Université Pierre et Marie Curie - CNRS-IRD-MNHN, LOCEAN/IPSLParis, France; ^2^Sorbonne Universités, Université Pierre et Marie Curie - UVSQ-CNRS, LATMOS/IPSLParis, France

**Keywords:** West African monsoon, climate change, impacts, adaptation, agriculture

## Abstract

West Africa is known to be particularly vulnerable to climate change due to high climate variability, high reliance on rain-fed agriculture, and limited economic and institutional capacity to respond to climate variability and change. In this context, better knowledge of how climate will change in West Africa and how such changes will impact crop productivity is crucial to inform policies that may counteract the adverse effects. This review paper provides a comprehensive overview of climate change impacts on agriculture in West Africa based on the recent scientific literature. West Africa is nowadays experiencing a rapid climate change, characterized by a widespread warming, a recovery of the monsoonal precipitation, and an increase in the occurrence of climate extremes. The observed climate tendencies are also projected to continue in the twenty-first century under moderate and high emission scenarios, although large uncertainties still affect simulations of the future West African climate, especially regarding the summer precipitation. However, despite diverging future projections of the monsoonal rainfall, which is essential for rain-fed agriculture, a robust evidence of yield loss in West Africa emerges. This yield loss is mainly driven by increased mean temperature while potential wetter or drier conditions as well as elevated CO_2_ concentrations can modulate this effect. Potential for adaptation is illustrated for major crops in West Africa through a selection of studies based on process-based crop models to adjust cropping systems (change in varieties, sowing dates and density, irrigation, fertilizer management) to future climate. Results of the cited studies are crop and region specific and no clear conclusions can be made regarding the most effective adaptation options. Further efforts are needed to improve modeling of the monsoon system and to better quantify the uncertainty in its changes under a warmer climate, in the response of the crops to such changes and in the potential for adaptation.

## Introduction

Climate has a strong influence on agriculture, considered as the most weather-dependent of all human activities (Hansen, 2002) with impacts on food security (Schmidhuber and Tubiello, 2007). Both variability and change in climate affect food production availability, stability of food supplies, food utilization, access to food and food prices everywhere in the world (Schmidhuber and Tubiello, 2007). It is especially true in Sub-Saharan Africa which is known to be particularly vulnerable to climate change due to a combination of naturally high levels of climate variability, high reliance on rain-fed agriculture and limited economic and institutional capacity to cope with and adapt to climate variability and change (Challinor et al., 2007; Müller et al., 2010; Roudier et al., 2011). Indeed, under its current climate Sub-Saharan Africa is already facing recurrent food crises and water scarcity triggered or exacerbated by climate variability and extreme events such as droughts, excessive rains and floods which affect agricultural productivity and hence rural household food security (Dilley et al., 2005; Haile, 2005). This chronic food insecurity may even increase in the future since the food demand is expected to be multiplied by more than five in Africa by 2050 (Collomb, 1999).

Climate change and its impact on food security are additional strains on the agriculture sector in Africa. The last Intergovernmental Panel on Climate Change (IPCC, 2014) highlighted that: “warming of the climate system is unequivocal, and since the 1950s, many of the observed changes are unprecedented over decades to millennia. The atmosphere and ocean have warmed, the amounts of snow and ice have diminished, and sea level has risen. Changes in many extreme weather and climate events have been observed since about 1950. Recent climate changes have had widespread impacts on human and natural systems.” Moreover, “continued emission of greenhouse gases will cause further warming and long-lasting changes in all components of the climate system, increasing the likelihood of severe, pervasive and irreversible impacts for people and ecosystems.” In this context, crop productivity, which is directly tied to climate variability, appears particularly exposed to current and future climate change impacts. Indeed, “many studies covering a wide range of regions and crops show that negative impacts of climate change on crop yields have been more common than positive impacts.” Moreover, “rural areas are expected to experience major impacts,” and “all aspects of food security are potentially affected by climate change, including food production, access, use, and price stability.” At the turn of the twenty-first century, West Africa has been identified among the primary observed climate change hot-spots, and among the most persistent and early emerging prominent hot-spots foreseen for the twenty-first century, because of the observed and projected widespread increase in mean temperature and extreme hot-season occurrence (Turco et al., 2015). Given the particularly strong deep connection between crop production and climate variability in West Africa since agriculture is mostly rain-fed and crop management (use of fertilizers and pesticides combined with modern cultivars) remains low (Dingkuhn et al., 2006), the detected sensitivity to recent and future climate change makes the region a hotspot even in terms of food production and security.

In the context described above, better knowledge of how climate will change in West Africa and how such changes will impact crop productivity is crucial to inform policies that may counteract the adverse effects. Furthermore, the ability to identify the most suitable crop varieties and practices with the most robust characteristics for withstanding climate change, is crucial for formulating adaptation strategies in this region where farmers are already able to select adapted varieties (e.g., late or early millet) or to adapt their practices (e.g., delayed or early sowing) to a changed environment (Dingkuhn et al., 2006). However, although there is a growing literature on the impact of climate change on crop productivity in Africa, there are large uncertainties in climate change projections, in the response of crops to such changes and in the adaptation of agricultural systems to future climate conditions (Challinor et al., 2007; Roudier et al., 2011). Thus, this paper provides a comprehensive overview of climate change impacts on agriculture in West Africa based on the recent scientific literature.

This review is based on a wide review of the literature on climate variability and change in West Africa and associated impacts on crop productivity. Given the sensitivity of the topic, the available literature is vast (more than 200 papers are cited in the references), the review presented here does not claim to be exhaustive and certainly misses many studies. However, an effort has been done to present a selection of the most important results, with a special attention to the recent studies. Moreover, the extensive and coordinated discussion of the crop productivity problem and the related climate dynamics aspects represents the noticeable novelty of this review. Section Climate Change Scenarios of this review paper provides observed evidences of climate change in West Africa and gives some robust features about expected changes in the next decades. Section The Impact on Crop Yield and Potential for Adaptation investigates how such climate changes affect crop production as well as potential for adaptation for the major crops in West Africa. Each section attempts to stress the most robust results in the screened literature but, more importantly, includes a discussion about limitations and uncertainties. The reader is invited to read the cited papers for more details on any specific aspects discussed in this review.

## Climate change scenarios

### West african climate and monsoon dynamics

The West African climate is deeply tied to the West African monsoon (WAM) system, which develops in May over the Guinean coast (~5–10°N), reaches the maturity in August in the Sahel (~10–15°N), and finally retreats to the coast in October (Sultan and Janicot, 2003; Cook, 2015), concentrating in this period more than 70% of the annual precipitation in the region (CLIVAR, 2015). The monsoonal rainfall is a key element of the regional climate, especially in the semiarid Sahel, where vegetation is highly sensitive to precipitation variability, at time scales from intraseasonal to interannual (Philippon et al., 2007; Martiny et al., 2010; Taylor, 2011). Moreover, the atmospheric circulation characterizing the monsoonal system is associated with mineral dust emission (Bou Karam et al., 2007; Wang et al., 2015) and thermal anomalies (Guichard et al., 2009; Fontaine et al., 2013) in the region.

The WAM is the response to the land-sea thermal contrast triggered by the seasonal cycle of incoming insolation at the surface, which favors the inland penetration of the deep convection associated with the intertropical convergence zone (ITCZ; Thorncroft et al., 2011). In the lower troposphere, the atmospheric circulation is characterized by a southwesterly moist flow from the Gulf of Guinea, contrasting a dry northeasterly flow crossing the Sahara desert. This intertropical front can be regarded as the northern boundary of the WAM, and at the peak of the monsoonal season it is displaced around 20°N (Issa Lélé and Lamb, 2010). In the mid troposphere, the circulation is dominated around 12°N by the African easterly jet, originated by the meridional thermal gradient between the vegetated Guinean coast and the Sahara desert (Thorncroft and Blackburn, 1999). The African easterly jet is the wave guide for synoptic disturbances propagating westward along the Guinean coast and the Sahelian belt, known as African easterly waves (Poan et al., 2015). These disturbances are particularly important in triggering the monsoonal precipitation through the initiation and organization of mesoscale convective systems and squall lines during the monsoonal season (Cretat et al., 2015). The annual evolution of the WAM thermodynamic features (moisture fluxes and convergence), and of the associated rainfall distribution, is strongly impacted by the emergence of the Atlantic cold tongue, and the installation of the Saharan heat low. The Atlantic cold tongue is a cold pool which characterizes the equatorial eastern Atlantic Ocean from boreal spring to early summer, and its variability influences the timing of the monsoon onset over the Guinean coast and the intensity of the inland precipitation (Druyan and Fulakeza, 2015). The Saharan heat low is a lower tropospheric thermal depression over the Sahara desert west of 10°E, developing in response to the surface heating over West Africa in boreal summer (Lavaysse et al., 2009). The Saharan heat low onset is closely linked to the WAM onset in late June, and its variability modulates the longitudinal distribution of the monsoonal precipitation in the Sahel, being strong Saharan heat low phases associated with wet/dry anomalies in eastern/western Sahel (Lavaysse et al., 2010).

### Multi-time scales variability

In the twentieth century, the West African climate has been characterized by the variability of the WAM, showing a succession of long lasting wet and dry periods. This climate variability has been particularly relevant in the Sahel, where a large scale drought during the 70s–80s has been followed by a partial recovery of precipitation at the turn of the twenty-first century (Trenberth et al., 2007). The main driver of the WAM variability at time scales from intraseasonal to multidecadal is the global ocean sea surface temperature (SST; Pomposi et al., 2015; Rodríguez-Fonseca et al., 2015).

The observed 40-day variability of the WAM is mainly related to SST anomalies in the Indian Ocean associated with the Madden-Julian oscillation, which trigger convection disturbances traveling along the Equator and modulating the WAM precipitation (Pohl et al., 2009; Mohino et al., 2012).

The SST variability in the Tropical Atlantic is the main driver of the monsoonal circulation at the interannual time scales, through the land-sea thermal gradient which influences the meridional displacement of the precipitation belt, with the strongest impact on the Guinean coast (Polo et al., 2008; Losada et al., 2010). The Mediterranean Sea plays a role in modulating the interannual variability of the monsoonal precipitation over the Sahel, by feeding the convergence over the Sahel with moisture transported across Sahara (Fontaine et al., 2010; Gaetani et al., 2010). The WAM interannual variability is also remotely influenced by the SST variability in the Tropical Indian/Pacific Oceans, which may induce stationary waves propagating along the Equator and interacting over the Sahel (Rowell, 2001; Mohino et al., 2011b). These regional and remote connections are not stationary and are modulated at decadal and multidecadal time scales (Fontaine et al., 2011a).

The multidecadal variability of the WAM dynamics results from the combination of diverse low frequency global ocean signals (Mohino et al., 2011a). On the one hand, the warming of the Tropical Ocean, associated with global warming and positive phases of the interdecadal Pacific oscillation, favors dry conditions in the Sahel, through the inhibition of the tropical convection (Bader and Latif, 2003; Villamayor and Mohino, 2015). On the other hand, positive phases of the Atlantic multidecadal variability, by displacing northward the ITCZ, favor precipitation in the Sahel (Zhang and Delworth, 2006; Ting et al., 2009). The severe drought that affected the Sahel during the 70s–80s has been attributed to a negative Atlantic multidecadal variability phase, concomitant with a positive interdecadal Pacific oscillation phase, in a global warming context (Mohino et al., 2011a).

Other than to the SST forcing, the West African climate is highly sensitive to land surface conditions and processes. Vegetation-associated land surface processes have in West Africa the largest climate impact worldwide, especially in summer (Ma et al., 2013), and the Sahel shows the strongest soil moisture/climate coupling (Koster et al., 2006). In this context, it has been shown that the vegetation degradation has a role in the drought events in the Sahel, through the increase in albedo and the reduction of evaporation, leading to reduced net radiation and inhibited convection, respectively, which in turn weaken the monsoonal circulation (Xue, 2004).

### Modeling the west African climate

In the last 15 years, a big effort has been made to understand climate variability and change in West Africa. The African Monsoon Multidisciplinary Analysis program (AMMA; http://amma-international.org/), launched in 2002 and involving a number of research institutions in the international scientific community, was the first large scale coordinated program aiming to improve the understanding of the WAM system and its influence on the physical, chemical and biological environment, regionally, and globally. The AMMA community is still active to provide the underpinning science to assess the impacts of WAM variability on health, water resources, food security and demography in the West African countries, and to define and implement monitoring and prediction strategies (Redelsperger et al., 2006). Specifically addressed to climate modeling issues, the WAM Modeling and Evaluation project (WAMME; Druyan, 2011) is an initiative designed to evaluate the performance of global and regional climate models (GCMs and RCMs, respectively) in simulating the WAM dynamics and associated precipitation.

In the context of the Coupled Model Intercomparison Project Phase 3 and 5 (CMIP3 (Meehl et al., 2007) and CMIP5 (Taylor et al., 2012), respectively, a World Climate Research Programme (WCRP, http://www.wcrp-climate.org/) standard experimental protocol for studying the output of coupled atmosphere-ocean GCMs, climate variability in West Africa is extensively studied, with promising but still unsatisfying results. Specifically, state-of-the-art climate models in both CMIP3 and CMIP5 exercises show low skill in simulating the observed WAM variability (amplitude, phases, and trends), and sizable uncertainties affect projections in the twenty-first century, ranging from dry to wet conditions in the Sahel (Biasutti, 2013). Although coupled models generally well reproduce the relationship between the regional atmospheric circulation and the monsoonal precipitation, during both the twentieth and the twenty-first century, the same models show discrepancies in future projections (Biasutti et al., 2009). Therefore, model shortcomings can be firstly related to the ability in reproducing the large scale mechanisms which influence the regional atmospheric circulation, and especially the teleconnections with the global SST teleconnections (Biasutti et al., 2009; Rowell, 2013). An important source of uncertainty in the modeling of climate change in West Africa is also the model responses to the direct and indirect CO_2_ radiative forcing in the atmosphere: the former rapidly warms the continental surface, inducing a positive response in the WAM precipitation; the latter slowly warms the ocean surface, inducing dry conditions (Giannini, 2010). It has been shown that wet and dry model biases over West Africa may be related to an unbalanced model response to the direct and indirect CO_2_ forcing (Gaetani et al., 2016). At a regional scale, limitations in the model representation of SST in the Tropical Atlantic (Roehrig et al., 2013), surface heat fluxes (Xue et al., 2010), vegetation feedback (Kucharski et al., 2013), land use (Bamba Sylla et al., 2016), and mineral dust atmospheric concentration (Tompkins et al., 2005) are sources of incorrect simulations of the temporal and spatial variability of the WAM precipitation. Finally, the coarse resolution typical of GCMs limits the model ability to simulate the intense and organized convection characterizing the WAM (Vellinga et al., 2016). The assessment of model performances is critical to understand the sources of errors and limit uncertainties, but an overall and objective evaluation is a particularly difficult task, because results may differ depending on the specific variable analyzed and the metrics used. In the CMIP5 archive, a discrimination in the model performances for the historical climate may be achieved, but uncertainty in the projections is not reduced when skillful models are selected (Rowell et al., 2016). This suggests that the underlying assumption relating the model shortcomings in simulating past, present and future climate in West Africa is incorrect, being the assumption that the same modeled processes lead to errors in the simulation of the historical climate and uncertainty in projected change (Rowell et al., 2016). Therefore, further research, based on the understanding of the mechanisms that drive the errors and uncertainty in projected changes, is needed to discriminate model performances.

In the CMIP5 exercise, a specific effort had been devoted to climate prediction at decadal time scales (10–30 years), which is recognized as a key planning horizon in a socioeconomic perspective (Doblas-Reyes et al., 2013). Results demonstrate that the WAM variability at decadal time scales is influenced by both the global SST natural variability and the green-house gases (GHG) external forcing, and the prediction skill is highly model dependent (Gaetani and Mohino, 2013; Martin and Thorncroft, 2014; Otero et al., 2015). Specifically, highest skill models are characterized by the ability in reproducing the WAM connection with, primarily, the Atlantic multidecadal variability (Gaetani and Mohino, 2013) and, secondly, with the relative SST difference between the subtropical North Atlantic and the tropics and Mediterranean SST (Martin and Thorncroft, 2014).

In the framework of the Coordinated Regional Climate Downscaling Experiment (CORDEX, http://www.cordex.org/), a WCRP initiative for the assessment and comparison of RCM skills in diverse regions, CORDEX-Africa provides a set of state-of-the-art simulations and predictions for the West African climate at high resolution (Nikulin et al., 2012). The availability of reliable climate simulations at high spatial-temporal resolution is crucial for a robust assessment of climate impacts at regional scale, and the CORDEX-Africa exercise shows encouraging results for West Africa. The dynamical downscaling of GCMs, operated at higher resolution by the RCMs, leads to improvements in the simulation of the atmospheric circulation, temperature and precipitation climatology, as well as the occurrence of wet and dry spells, the frequency of heavy rain events, and the drought geographical distribution (Laprise et al., 2013; Bucchignani et al., 2016; Buontempo et al., 2015; Diasso and Abiodun, 2015; Dosio et al., 2015), although the biases in the lateral boundary conditions provided by the driving GCMs may significantly affect the RCMs outputs (Laprise et al., 2013; Dosio et al., 2015). Being the GCM biases more pronounced over the Tropical Atlantic, the RCM performances are in general better over the Sahel than in the Guinean coast, which is more influenced by the local SST variability (Paxian et al., 2016). Uncertainties in the simulation of daily precipitation are also observed, mainly related to the diverse convection schemes utilized in the CORDEX-Africa models (Klutse et al., 2016). However, the spread in the individual model performances is substantially improved when the ensemble mean is computed (Klutse et al., 2016).

### Recent climate change

After the devastating drought of the 70s–80s, West Africa is nowadays experiencing a partial recovery of precipitation, with a coherent increase in the annual rainfall in the Sahel (29–43 mm/year per decade in the period 1983–2010; Maidment et al., 2015). This recovery is characterized by a modification of the seasonal cycle, showing a delay of the monsoon retreat in the Sahel (two days per decade in the period 1983–2010; Sanogo et al., 2015), and by a change in the rainfall regime, showing a decrease in the number of rainy days and an increase in the proportion of annual rainfall associated with extreme events (17% in the period 1970-1990 and 21% in the period 2001–2010; Panthou et al., 2014). This precipitation recovery is accompanied by a stable rainfall/vegetation trend (Hoscilo et al., 2015). The recent climate change is also characterized by modifications in terms of atmospheric circulation and surface temperature. The meridional overturning cell associated with the monsoonal circulation is shifted ~1° northward, with changes in the convection belt in West Africa and the subsidence over the Mediterranean region (Fontaine et al., 2011b). Moreover, an amplified warming of the Sahara desert is detected (Cook and Vizy, 2015), and the Saharan heat low shows an intensification (Lavaysse et al., 2015) with reduced desert dust emission in summer (Wang et al., 2015). The origin of this climate change signal in the Sahara region has been related to the direct radiative forcing of the increased CO_2_ concentration (Gaetani et al., 2016) and to an augmented moisture availability in the lower troposphere over the desert, triggering a water vapor-temperature feedback (Evan et al., 2015). The changes in the regional atmospheric dynamics accompanies positive temperature anomalies and extremes in spring and summer in the Sahel (Fontaine et al., 2013; Russo et al., 2016). Using a network of 90 *in situ* observations in West Africa, Moron et al. (2016) found that the linear trends of annual mean maximum and minimum temperature equal respectively +0.021°C/year and +0.028°C/year.

The debate on the origin of the recent precipitation recovery in West Africa and the associated modifications in the regional atmospheric dynamics is open and heated, and the positions may be conveyed into two main arguments. On the one hand, the recovery is ascribed to the northward migration of the ITCZ in response to the SST warming at end of the twentieth century, which was stronger in the Northern Hemisphere than in Global Tropical Ocean (Park et al., 2014). A role of the warming of the subtropical North Atlantic in providing the moisture to feed the monsoonal system has been identified (Giannini et al., 2013). On the other hand, a dominant role of the direct GHG radiative forcing is hypothesized, acting by warming the surface and increasing evaporation over the continental surface (Dong and Sutton, 2015).

### Future projections

In the CMIP5 exercise, a positive trend in the WAM precipitation results from the multi-model mean in the twenty-first century, though the individual model projections are characterized by a large spread (Biasutti, 2013). Indeed, about 50% of the model runs in the CMIP5 archive shows a robust positive trend, about 25% shows a robust decreasing trend, while the trend is negligible in the remaining 25% (Biasutti, 2013). In the models predicting wet conditions, these are related to the direct radiative effect of the increase in GHG concentration, leading to local increased evaporation and vertical instability (Hoerling et al., 2006; Giannini, 2010). On the contrary, models projecting dry conditions simulate reduced moisture transport and deep convection over land as a response to the global ocean warming, which heats the troposphere and imposes stability (Held et al., 2005; Caminade and Terray, 2010). Therefore, the competition between the response of the land-atmosphere system to the local GHG radiative forcing, and the response mediated through the warming of the global SST, emerges as a key component of the West African climate change (Bony et al., 2013; Gaetani et al., 2016), and understanding the relative impact of these two diverse forcings represents a task of primary importance for the climate modeling community.

**Figure 1:**
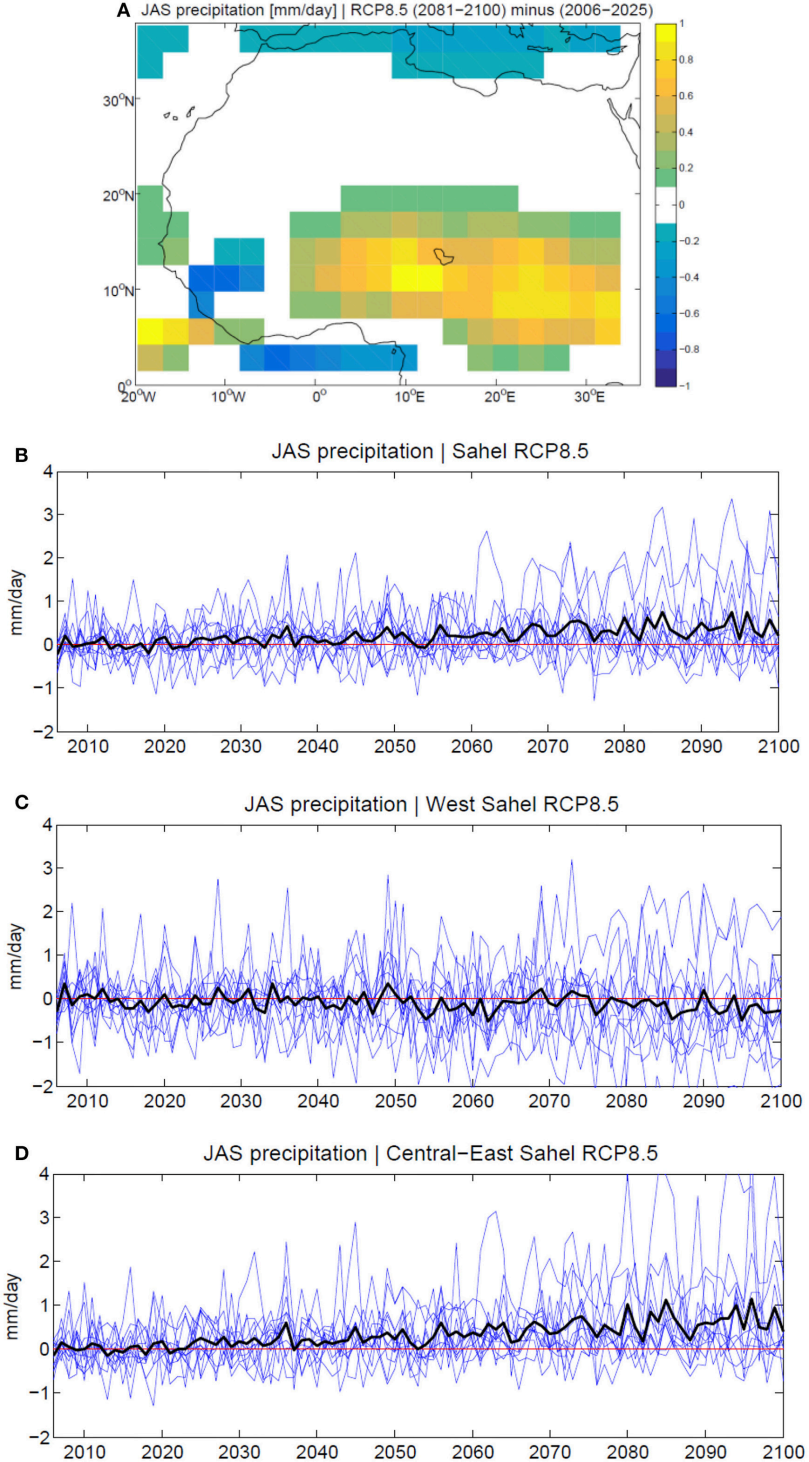
**WAM precipitation evolution in the twenty-first century, simulated by 12 CMIP5 models in the RCP8.5 scenario (van Vuuren, 2011). (A)** Projected change in multi-model mean of the July-to-September (JAS) precipitation [mm/day] at the end of the twenty-first century (2081-2100), represented by computing the difference with the period 2006-2025. Significance is estimated through a Student's *t*-test at 90% level of confidence. Time series of the WAM precipitation averaged in **(B)** Sahel [15°W-30°E, 7-20°N], **(C)** western Sahel (west of 5°W) and **(D)** central-eastern Sahel (east of 5°E). The twenty-first century anomalies are computed regarding the period 2006-2015. The models analyzed are: BCC-CSM1-1, CanESM2, CCSM4, CNRM-CM5, FGOALS-g2, HadGEM2-CC, IPSL-CM5A-LR, IPSL-CM5B-LR, MIROC5, MPI-ESM-LR, MPI-ESM-MR, MRI-CGCM3. For data availability and accessibility, the reader may refer to the CMIP5 web portal at http://cmip-pcmdi.llnl.gov/cmip5/availability.html.

The future projection in precipitation simulated by climate models in the twenty-first century is not spatially homogeneous over the Sahel. Indeed, future wet conditions in central-eastern Sahel (east of ~0°E) contrast with dry anomalies over western Sahel (west of ~0°E), and these sub-regional trends are more robust than the trend simulated in the extended Sahelian belt (Monerie et al., 2012, 2013; Biasutti, 2013). The rainfall excess expected in central-eastern Sahel is mainly linked to a strengthening and northward shift of the meridional overturning circulation over West Africa, reinforcing the monsoonal flow, with a feedback in the lower levels from the increased temperature and evaporation associated with the GHG radiative forcing (Monerie et al., 2012). The projected dry spot over western Sahel is associated with a reinforcement of the African easterly jet and modifications in the overturning zonal circulation connecting the Indian and Atlantic Oceans, which result in anomalous subsidence on its descending branch over subtropical North Atlantic (Monerie et al., 2012). Moreover, this east-west anomaly dipole in precipitation is consistent with the recently observed long term intensification of the Saharan heat low (Lavaysse et al., 2015). The projected rainfall trends result to be gradually enhanced and extended in future scenarios with a global warming of 2–4°C and beyond, showing an approximately linear amplification with no tipping points being reached (James and Washington, 2013; James et al., 2014). The twenty-first century evolution of the WAM precipitation simulated by a subset of the CMIP5 models is illustrated in Figure 1.

The WAM seasonal cycle is also affected by climate change in the twenty-first century. The projected precipitation increase in the central-eastern Sahel is characterized by a robust increase of the rainfall amounts in September-October (70% of the CMIP5 model runs; Biasutti, 2013). This results in a delay of the monsoon withdrawal, with a lengthening of the monsoon season (Monerie et al., 2016). The moisture transport dominates the water budget change in September, while the local recycling role is prominent in October (Monerie et al., 2016). Conversely, the drying of the western Sahel appears to be concentrated in June-July in 80% of the CMIP5 model runs (Biasutti, 2013). The future modifications in the WAM seasonal cycle are accompanied by coherent changes in the African easterly wave activity, showing a reduction in late spring and early summer and a large increase between July and October, although large differences exists in African easterly wave projections between high- and low-resolution models (Skinner and Diffenbaugh, 2014; Martin and Thorncroft, 2015).

In contrast to the uncertainties affecting the future projection of the West African rainfall, a broad consensus characterizes the model simulations of the surface temperature for the twenty-first century. The future change in the monsoonal regime will be accompanied by a general warming of the African continent, with a maximum over the Sahara desert, ranging between 3 and 7°C, depending on the model and the emission scenario (Monerie et al., 2012; Dike et al., 2015). Boreal winter in West Africa will be also affected by a 2–3°C warming, with the strongest anomalies over the Guinea coast (Dike et al., 2015).

High resolution RCMs provide a detailed description of the future climate change in West Africa, generally agreeing with GCMs on the temperature projection in the region. A robust warming is predicted throughout the twenty-first century, although even large differences (more than 1°C) with the driving GCMs exist locally (Laprise et al., 2013; Dosio and Panitz, 2016). This will be accompanied, in the mid-twenty-first-century, by an increase in the number of heat wave days, by 20–120 days per year over the Sahel, by 20–60 days over Western Sahara, and by 5–40 days over eastern Sahara (Vizy and Cook, 2012). Moreover, half of the CORDEX-Africa projections suggests that heat waves that are unusual under present climate conditions in West Africa, will occur on a regular basis by 2040 under high emission scenarios (Russo et al., 2016). Finally, in the mid-twenty-first-century, daily maximum and minimum temperatures are projected to increase, and the daily diurnal temperature range to decrease, by 0.3–1.2°C during boreal spring and fall over West Africa, and by 0.5–1.5°C during boreal summer over the Sahel (Vizy and Cook, 2012).

The number of dry days is predicted to decrease by 3–7% over central Africa in spring and over eastern Sahel in summer. Conversely, the occurrence of extreme wet days will increase over West Africa by 40–60% (1–4 days) and the southern Sahel by 50–90% (1–4 days), uniformly during boreal summer. The associated changes in extreme wet rainfall intensity show a regional response, including a 30–70% decrease over northern Niger and northeastern Mali, and a 10–25% increase over Senegal, southern Mali, Burkina Faso, northern Nigeria, and southern Chad (Vizy and Cook, 2012). However, future RCM rainfall projections are affected by large uncertainties. On the one hand, RCMs tend to inherit the biases of the driving GCMs, so that a RCM downscaling several GCMs reproduces the inter-GCM spread, though with a reduced amplitude (Buontempo et al., 2015; Dosio and Panitz, 2016). On the other hand, a RCM may project its own trend regardless the inter-model spread of the driving GCMs, due to the differences in the specific physical formulation of RCMs and GCMs (Laprise et al., 2013; Buontempo et al., 2015; Saini et al., 2015).

Finally, it has been recently pointed out that the projected modification in the atmospheric dynamics over North Africa may impact the Saharan dust emission and atmospheric concentration, leading to a significant negative trend in the twenty-first century (Evan et al., 2016). Other than on human health in the region, expected to be benefitted, the reduction in dust concentration may have a positive feedback on the monsoonal precipitation, through a reduction in the associated surface cooling and lower troposphere heating, favoring atmospheric instability (Yoshioka et al., 2007; Ji et al., 2016).

## The impact on crop yield and potential for adaptation

### Predicting crop yield from GCM simulations

#### Crop models

Predicting the potential impacts of climate change on crop yields requires a model of how crops respond to future conditions induced by anthropogenic climate change, such as: warmer temperatures, more frequent extreme temperatures, possible changes in rainfall mean, seasonality spatial and temporal distribution. In addition, there is a direct impact of atmospheric composition on crops with elevated levels of carbon dioxide acting to increase crop yields through the stimulation of photosynthesis and reduction of drought stress (Tubiello et al., 2007; Leakey, 2009) while elevated levels of atmospheric ozone which are expected in developing countries like Africa (Royal Society, 2008) can lead to yield losses (Van Dingenen et al., 2008). Crop models typically simulate the response of the crop to variability and change in weather and climate related to temperature, precipitation and radiation, and atmospheric CO_2_ concentration (Ewert et al., 2015). There are numerous crop models with different levels of sophistication (Di Paola et al., 2016) and several reviews can be found in the literature, describing the concepts and limitations (see for instance Boote et al., 1996; White et al., 2011; Affholder et al., 2012; Ewert et al., 2015; Di Paola et al., 2016). Crop models can be roughly divided into two categories: statistical models trained on historical yields and some simplified measurements of weather, such as growing season average temperature and precipitation (Lobell and Burke, 2009) and process-based crop models which simulate explicitly the main processes of crop growth and development (see for instance Ewert et al., 2015). Table 1 shows a selection of models that have been used to assess the impact of climate change on yields of various crops in West Africa. If the use of process-based models for climate change impact and risk assessment studies has become increasingly important (Tubiello and Ewert, 2002; Challinor et al., 2009; White et al., 2011; Rötter et al., 2012; Angulo et al., 2013; Ewert et al., 2015) since they are able to simulate impacts of climate, CO_2_ concentrations on bio-physical processes (e.g., phenology, photosynthesis, respiration, transpiration, and soil evaporation) and other production constraints such as N limitations, these models require extensive input data on cultivar, management, and soil conditions as well as calibration and validation data that are often unavailable in Africa (Lobell and Burke, 2010). Even in the presence of such data these models can be very difficult to calibrate because of a large numbers of uncertain parameters (Iizumi et al., 2009; Tao et al., 2009). Furthermore, research effort in crop modeling has focused on the world's major food crops such as wheat, maize, rize, and sorghum and the simulation of crops common in African farming systems (sorghum, millets, yam) is less well developed as well as simulations of crops grown as intercrops across Africa (Challinor et al., 2007; White et al., 2011). Ensemble modeling including a variety of crop models is thus highly recommended to enable a quantification of the uncertainty (Challinor et al., 2009). In this context, extensive model intercomparisons such as the ones conducted throughout the Agricultural Model Intercomparison and Improvement Project (AgMIP; http://www.agmip.org/; Rosenzweig et al., 2014), which includes Sub-Saharan Africa as one of the target region (Adiku et al., 2015), are likely to improve substantially the characterization of the threat of crop yield losses and food insecurity due to climate change.

**Table 1:** **A selection of crop models (including combination between crop models) that have been used to assess the impact of climate change on yields of various crops in West Africa in the recent scientific literature**.

**Crop model**	**Area**	**Crop**	**References**
EPIC	Nigeria	Cassava, maize, millet, rice, sorghum	Adejuwon, 2006
Empirical	Niger	Millet	Ben Mohamed et al., 2002
EPIC + PHYGROW + NUTBAL	Mali	Cotton, cowpea, groundnut, maize, millet, sorghum	Butt et al., 2005
AEZ + BLS	Sub-Saharan Africa	Global	Fischer et al., 2005
IMPACT + DSSAT	Sub-Saharan Africa	Global, maize, millet, rice, sorgum, wheat, soybean, groundnut	Nelson et al., 2009
CERES − maize	West Africa	Maize	Jones and Thornton, 2003
CERES − maize + Empirical	Niger, Nigeria, Mali, Guinea, Ivory Coast, Cameroun	Maize	Lobell and Burke, 2010
GEPIC	Sub-Saharan Africa, West Africa	Global, cassava, maize, millet, rice, sorghum, wheat	Liu et al., 2008
Empirical	West Africa	Cassava, groundnut, maize, millet, rice, sorghum, wheat, yams	Lobell et al., 2008
LPJmL	West Africa	Global	Müller et al., 2010
MOS (empirical)	Benin	Beans, cassava, cotton, groundnut, maize, rice, sorghum, yams	Paeth et al., 2008
Empirical + BLS	West Africa	Global	Parry et al., 2004
DSSAT	Niger, Burkina Faso	Millet (two cultivars), sorghum	Salack, 2006
Empirical	West Africa	Cassava, groundnut, maize, millet, sorghum	Schlenker and Lobell, 2010
DSSAT	Gambia	Groundnut, maize, millet late, millet early	Smith et al., 1996
Cropsyst	Cameroon	Bambara nut, groundnut, maize, sorghum, soybean	Tingem and Rivington, 2009
Empirical	Niger	Cowpea, groundnut	Vanduivenbooden et al., 2002
SARRA-H + APSIM	West Africa	Sorghum (two cultivars)	Sultan et al., 2014
SARRA-H	West Africa	Millet (three cultivars), Sorghum (three cultivars)	Sultan et al., 2013
CROPGRO	Cameroon	Cotton	Gerardeaux et al., 2013
EPIC + GEPIC + LPJ-GUESS + pDSSAT + PEGASUS	Burkina Faso, Senegal	Maize, Wheat, Soybean, Rice, Millet, Sorghum, Sugarcane, Beans, Cassava, Cotton, Sunflower, Groundnut	Deryng, 2015
SARRA-H + EPIC	Niger, Benin	Maize, Millet	Ramarohetra et al., 2015
DSSAT	Niger	Millet	Rezaei et al., 2014
EPIC	Benin	Yam (early and late cultivars)	Srivastava et al., 2015
EPIC	Benin	Maize	Gaiser et al., 2010
ORCHIDEE	West Africa	C4 crop	Berg et al., 2013
GLAM	West Africa	Groundnut	Parkes et al., 2015
GEPIC	Sub-Saharan Africa	Maize	Folberth et al., 2014
DSSAT + APSIM	Senegal, Ghana	Maize, Millet, Peanut	Adiku et al., 2015
EcoCrop	Africa	Maize, millets, sorghum, banana, and beans	Jarvis et al., 2012

#### Link with climate

The use of climate projections from GCMs to force crop models is challenging and raises several important issues. First, combining GCMs and process-based crop models raises a scale mismatch since climate models typically operate on spatial scales much larger than the processes governing the yields at the plot scale and most factors affecting crops such as soil properties and farming practices (Baron et al., 2005; Challinor et al., 2009). To overcome this issue, climate data can be downscaled to the scale of a crop model with two types of downscaling approaches that can be sometimes combined (see for instance Zorita and von Storch, 1999). Statistical downscaling relies on the use of empirical relationships between mesoscale and local climate observed variables to relate GCM output to local climate (Zorita and von Storch, 1999). An alternative approach is the use of dynamical downscaling which offers a self-consistent approach that captures fine-scale topographic features and coastal boundaries by using regional climate models (RCMs) with a fine resolution (~10–50 km) nested in the GCM (Paeth et al., 2011; Glotter et al., 2014). The use of dynamical downscaling in long-range climate projections has recently increased with the growth of computing resources and large simulations databases of downscaled climate outputs are available for intercomparison and impacts assessment (Glotter et al., 2014). For instance the international Coordinated Regional Climate Downscaling Experiment Africa (CORDEX Africa) simulations are now publicly available and used in the literature, including a downscaled subset of GCMs simulations with different RCMs (Diallo et al., 2016). However, although it can improve weather and climate variability (Feser et al., 2011; Gutmann et al., 2012) as well as crop yield projections (e.g., Mearns et al., 1999, 2001; Adams et al., 2003; Tsvetsinskaya et al., 2003), it is important to keep in mind that downscaling is an additional source of errors and uncertainties to crop yield projections. For example, when different RCMs were used to downscale atmospheric re-analyses to force the SARRA-H crop model in Senegal, Oettli et al. (2011), large differences were found in the simulated sorghum yields depending on the RCM used. More recently, Ramarohetra et al. (2015) conducted a sensitivity analysis of the WRF model and found that a change in the physical parameterizations of a single RCM as well as internal variability of the RCM can lead to major changes in the simulation of crop yields of millet and maize in West Africa. As alternative to downscaling, the use of large-area crop modeling has grown in recent years (Challinor et al., 2004, 2009; Tao et al., 2009). This approach offers the possibility of using the outputs from climate models directly in a process-based way, suppressing the needs for downscaling, has grown in the literature (Challinor et al., 2004, 2009). Several models have been used in West Africa like the GLAM model used to simulate groundnut (Parkes et al., 2015) or LPJ-ml (Müller et al., 2010) and ORCHIDEE (Berg et al., 2011, 2013) which are part of Earth System vegetation models in which they account for tropical croplands.

The second issue raised by the use of GCM for assessing climate impacts is that climate models show significant biases in simulating current climate with sometimes insufficient skill for GCM outputs to be used directly as inputs for impact models without prior bias correction (Semenov and Barrow, 1997). If bias-correction is often included into statistical downscaling, the skill of representing the present-day climate can be very low using regional downscaling (Oettli et al., 2011). Since impact models ultimately rely on the accuracy of climate input data (Berg et al., 2010), the errors inevitably propagated into the combined climate/crop modeling (Oettli et al., 2011; Glotter et al., 2014; Ramarohetra et al., 2015). For instance, using two RCMs and the DSSAT-CERES-maize crop model over the United States, Glotter et al. (2014) showed that although the RCMs correct some GCM biases related to fine-scale geographic features, the use of a RCM cannot compensate for broad-scale systematic errors that dominate the errors for simulated maize yields. Moreover, Ramirez-Villegas et al. (2013) suggested that the use of raw GCM outputs can even affect the estimation of the climate change impact on crop yields by significantly under- or overestimate cropping system sensitivity by 2.5–7.5% for precipitation-driven areas and 1.3–23% for temperature-driven areas. Thus, careful evaluation of climate models using regional key drivers of crop yields (Berg et al., 2010; Ramirez-Villegas et al., 2013; Guan et al., 2015) is needed to make the best use of climate change simulations for impact research. Large errors have been found in the simulation of the WAM rainfall by climate models which usually suffer from too much drizzle and a large bias in rainfall frequency, large errors in simulating seasonal rainfall as well as an underestimation of the interannual variability which can subsequently bias simulated crop yield (Baron et al., 2005; Berg et al., 2010; Ramirez-Villegas et al., 2013; Guan et al., 2015). Significant biases have also been found CMIP5 simulations for mean temperature and diurnal temperature ranges in West Africa (Ramirez-Villegas et al., 2013). To overcome this issue, climate impact studies generally require some level of climate data bias correction. The simplest correction method is the delta method used by Müller et al. (2010) or Sultan et al. (2013) which consists to add a computed mean annual anomaly between future and current simulated climates of a given GCM to a current observation-based dataset. Promising results are obtained by Oettli et al. (2011) when applying a more complex bias correction technique (Michelangeli et al., 2009) to climate model outputs. In particular the authors showed that means and standard deviations of simulated yields of sorghum in Senegal are much more realistic with bias corrected climate variables than those using raw climate models outputs.

Another important issue which has already been discussed in Section Climate Change Scenarios is the large plausible range of future climate changes at the regional scale of West Africa. Although there are some robust features in climate change scenarios in the region (see Section Climate Change Scenarios), there is a wide spread in current climate model projections of regional rainfall changes over West Africa, especially with respect to summertime rainfall totals (Druyan, 2011) which are crucial for yields of staple food crops in West Africa (Berg et al., 2010; Guan et al., 2015). Up to now, using the largest number of GCMs from the CMIP5 ensemble of around 36 GCMs remains the best way to represent the range of climate futures in impact assessment. Knox et al. (2012) showed that increasing the number of climate models used to force crop models reduces the median range and outliers about the mean change in future yields. Important biases or underestimation of uncertainties can be expected from climate impact assessments based on subsets of CMIP datasets, and similarly from downscaled or bias-corrected datasets (like CORDEX) which are based on a restricted subset of GCMs. This point is illustrated by McSweeney and Jones (2016) who investigated how well the widely used Inter-Sectoral Impact Model Inter-comparison Project (ISI-MIP) subset of five CMIP5 models (see for instance Adiku et al., 2015) represent the plausible range of future climate changes. They found that the fraction of the full range of future projections captured by the ISI-MIP subset is sometimes very low depending on the variable, the season and the region especially for summer rainfall and temperatures in the Western part of West Africa (McSweeney and Jones, 2016).

### Assessing climate impacts

#### The overall signal

Although there is a growing literature on the impact of climate change on crop productivity in tropical regions, it is difficult to provide a consistent assessment of future yield changes because of large uncertainties in regional climate change projections, in the response of crops to environmental change (rainfall, temperature, CO_2_ concentration), in the coupling between climate models and crop productivity functions, and in the adaptation of agricultural systems to progressive climate change (Challinor et al., 2007; Roudier et al., 2011). These uncertainties result in a large spread of crop yield projections indicating a low confidence in future yield projections. As an example of the diversity of yield scenarios that have been produced, Roudier et al. (2011) found that the response of crop yield to climate in change in West Africa can vary from −50% to +90% in a selection of 16 publications. This range is even larger in the review made by Müller et al. (2010) which showed that projected impacts relative to current African production levels range from −100% to +168%. This range reflects the variety of regions, crops, climate scenarios and models and crop models chosen in the studies.

To identify the main sources of uncertainty and establish robust estimates of the aggregate effects of climate change on crop yields, meta-analyses were conducted at the global scale by Challinor et al. (2014) to contribute to the food security and food production systems chapter of the Fifth Assessment Report (AR5) of the IPCC and at the regional scale, including West Africa (Roudier et al., 2011; Knox et al., 2012). Meta-analyses that combine and compare results from numerous studies are widely used in epidemiology and medicine and can be a useful way of summarizing the range of projected outcomes in the literature and assessing consensus. The meta-analysis conducted by Challinor et al. (2014) used a data set of more than 1700 published simulations to evaluate yield impacts of climate change and adaptation which is the largest pool of data from diverse modeling studies ever used for a global synthesis of this kind (Rötter, 2014). The meta-analyses published by Knox et al. (2012) and Roudier et al. (2011) are based on a smaller data set (1144 and 347 published simulations respectively) but concern specific regions: Asia and Africa in database compiled by Knox et al. (2012) and only West Africa in the database compiled by Roudier et al. (2011). These latter two meta-analyses also include the response of relevant crops in Africa (maize, sorghum, millet, rice, cotton, cassava, groundnut, yam) while the meta-analysis conducted by Challinor et al. (2014) includes only major crops such as maize, rice and wheat; maize and rice being the only crops of the study grown in West Africa. Interestingly, while there are all based on different approaches and different samples, the three studies came out with similar conclusions on how climate change will affect crop yield in West Africa and how this response varies across the different assumptions and methodological choices. While the magnitude of the response of crop yield to climate warming scenarios varies considerably in the simulations reported by Challinor et al. (2014), Knox et al. (2012) and Roudier et al. (2011), the sign of the change is mostly negative with a mean yield reduction of −8% was identified in all Africa (Knox et al., 2012) and −11% in West Africa (Roudier et al., 2014). Maize was found to be the most affected crop in West Africa and in the Sahel by Knox et al. (2012). Without adaptation, the mean response of major crops (mostly maize and rice) to climate change depicted by Challinor et al. (2014) in tropical regions is a yield reduction. This robust yield loss is already significant at moderate levels of local warming (+2°C) but is more consensual and stronger in the second half of the century when the additional radiative forcing is amplified. If this negative impact on crop yield was already depicted in the previous IPCC report, it suggested such yield loss would only occur when exceeding 3–4°C local warming which might be due to an overestimation in previous studies of the yield benefits of enhanced atmospheric CO_2_ (Rötter, 2014).

Such robust evidence of future yield loss in West Africa also confirmed in previous review of the literature (Challinor et al., 2007; Kotir, 2010; Müller et al., 2010) can be surprising in regards to the diverging projections in a warmer climate of summer monsoon rainfall. This is because of the adverse role of higher temperatures in shortening the crop cycle duration and increasing evapotranspiration demand and thus reducing crop yields, irrespective of rainfall changes (Schlenker and Lobell, 2010; Roudier et al., 2011; Berg et al., 2013; Sultan et al., 2013). Potential wetter conditions or elevated CO_2_ concentrations hardly counteract the adverse effect of higher temperatures (Sultan et al., 2014) while dryer conditions can strongly amplify the yield losses (Schlenker and Lobell, 2010; Roudier et al., 2011; Sultan et al., 2013, 2014).

#### Crop model differences

The response of the crop to climate change is subject to uncertainty that can arise from several sources (Challinor et al., 2009). In particular, significant differences were found in yield response from process-based vs. statistical models. Knox et al. (2012) and Roudier et al. (2011) both found that the dispersion around the mean is greater using process-based crop models. Furthermore, Challinor et al. (2014) found that statistical models predict a greater negative impact of climate on crop yields. The review of Müller et al. (2010) based on recent climate change impact assessments (14 quantitative, six qualitative) in Africa also stressed this larger dispersion with projected impacts relative to current production levels range from −84% to +62% in process-based and from −57% to +30% in statistical assessments. The larger dispersion of process-based crop models can be induced by the fact that they incorporate more complex factors in the yield response to climate change (CO_2_ effect, rainfall distribution, extreme temperatures) but also that the lack of sufficient data for accurate calibration and validation (Lobell and Burke, 2010; Lobell et al., 2011) and site specific parametrization of the crop management options and cultivars (Müller et al., 2010) in developing countries such in as Africa increase uncertainty in the crop response. More recently, systematic intercomparison studies of climate change impacts in West Africa were conducted using five process-based crop models (EPIC, GEPIC, LPJ-GUESS, pDSSAT, and PEGASUS; see Deryng, 2015) and two process-based crop models (DSSAT and APSIM in Adiku et al., 2015; SARRA-H and APSIM in Sultan et al., 2014) using the same forcing climate datasets. They all found a general agreement in the sign of the crop yield response to climate change scenarios while the amplitude of the impact varied strongly across models and simulated crops.

#### Regional differences

Important regional differences have been found in the response of crop yield to climate change. Roudier et al. (2011) found that cropped areas in the Soudano-Sahelian zone are likely to be more affected by climate change than those located in the Guinean zone. This difference can be explained by the projections of future climate in Africa which show a greater warming over continental Africa (particularly in the Sahel and Sahara) while the temperatures of the Guinean zone, which are influenced by the Atlantic Ocean, are expected to increase more slowly.

Using simulations of nine bias-corrected CMIP5 climate models and two crop models (SARRA-H and APSIM), Sultan et al. (2014) found a West-East dipole in the impacts of crop yield to climate change in West Africa. Indeed, in broad agreement with the full CMIP5 ensemble, their subset of bias-corrected climate models depicted a robust change in rainfall in West Africa with less rain in the Western part of the Sahel (Senegal, South-West Mali) and more rain in Central Sahel (Burkina Faso, South-West Niger) in the decades of 2031–2060 compared to a baseline of 1961–1990. In response to such climate change, but without accounting for direct crop responses to CO_2_, mean crop yield of sorghum decreases by about 16–20% and year-to-year variability increases in the Western part of the Sahel, while the eastern domain sees much milder impacts. This West-East dipole is confirmed by the study of Deryng (2015) which uses a set of five global climate models and six different global gridded crop models to assess climate change impacts on crop productivity in semi-arid croplands by the 2030s under the RCP 8.5 scenario. Without including the effect of elevated CO_2_ on crop photosynthesis and water demand, the author shows in Senegal, where three over five GCMs simulate drier conditions a median decrease of rainfed crop (−8.5 ± 9.9%) while in the Eastern part of West Africa in Burkina Faso, where four of the five GCMs simulate wetter conditions, the results show a slight decrease (−3.9 ± 4.3%). This dipole was also found in the study of Adiku et al. (2015) which used DSSAT and APSIM to simulate climate change impacts on crop yields in two locations in Nioro (Senegal) and Navrongo (Ghana). The effect of climate change was higher in the Senegalese site than in the one in Ghana using both crop simulation models.

#### The effect of elevated CO_2_

If rising atmospheric CO_2_ concentrations directly contributes to climate change, it has the potential to increase crop water productivity by enhancing photosynthesis and reducing leaf-level transpiration of plants (Tubiello et al., 2007; Leakey, 2009; Deryng et al., 2016). Significant increases of crop yield due to elevated levels of CO_2_ have been reported in experiments for different crops (Kimball, 1983; Kimball et al., 2002) and most of the recent modeling studies simulate the effect of elevated CO_2_ (Deryng et al., 2016). However, there is an ongoing debate about the extent of impacts of CO_2_ fertilization on crop yields in observations and models (Long et al., 2006; Ainsworth et al., 2008), especially in Africa where few field observations are unavailable to validate and further improve the models. In particular there is no free air carbon dioxide enrichment (FACE) experiments in Africa. Yet, the impact of higher atmospheric CO_2_ concentration is a major source of uncertainty in crop yield projections (Soussana et al., 2010; Roudier et al., 2011). For instance, by conducting a systematic comparison between yield response to climate change with, or without, CO_2_ fertilization effect, Müller et al. (2010) found a yield increase of 8% in Africa (percent change in 2046–2055 relative to 1996–2005) with full CO_2_ fertilization, and a yield loss of −8% without the CO_2_ effect. More recently, Deryng (2015) found that simulated median yield of rain-fed crops in six countries of semi-arid areas (including Senegal and Burkina Faso in West Africa) increases by 4.7 ± 9.6% when including the effects of both climate change and elevated CO_2_ concentrations while median yield decreases by 4.5 ± 7.3% when excluding the effects of elevated CO_2_ concentrations. Sultan et al. (2014) also found that CO_2_ fertilization would significantly reduce the negative climate impacts, increasing sorghum yields on average by 10%, and drier regions would have the largest benefits. However, other studies show lower differences between full and no CO_2_ fertilization scenarios (Berg et al., 2013). Overall most studies conclude that benefits of elevated CO_2_ will be greater for C3 crops (e.g., soybean, groundnut) which are likely to accumulate more biomass and for C4 crops in arid regions through increased water use efficiency (Berg et al., 2013; Sultan et al., 2014; Deryng et al., 2016). However, while showing benefits of higher CO_2_ concentrations on water crop productivity, Deryng (2015) and Sultan et al. (2014) both show that it partially offsets the impacts from climate changes especially in the Western part of Africa where yield losses are expected even after accounting for CO_2_ fertilization effect. Deryng (2015) found a decrease of crop yield of groundnut, millet, sorghum, and maize in Senegal by the 2030s even when including the effects of CO_2_. The author also found a slight increase of crop yield of millet and sorghum in Burkina Faso when including CO_2_ but yield of groundnut and maize decreases. Moreover, even if we can expect benefits from increasing CO_2_ on crop productivity, nutritional value may nevertheless be compromised (Müller et al., 2014). Indeed, a meta-analysis conducted by Myers et al. (2014) demonstrated that CO_2_ fertilization is likely to have adverse effects on the nutritional value of many key food crops by reducing the concentrations of essential minerals and protein with potential serious consequences in food security (Müller et al., 2014).

### Adaptation studies

Despite large uncertainty, there is a robust conclusion from the above section: agriculture in West Africa is at risk to be negatively affected by climate change. These potential adverse negative climatic changes effects are superimposed on top of high natural variability in seasonal rainfall, which historically has produced large inter-annual variations in rainfall and prolonged droughts (Giannini et al., 2008) and the recent increase in rainfall intensity and extreme heavy-rainfall events (Panthou et al., 2014). Both climate variability and trend pose a challenge for the primarily rain-fed agriculture systems in West Africa. Since the 1970's, the largest food crises in Africa that required large-scale external food aid (1974, 1984/1985, 1992, and 2002) have been attributed fully or partially to extreme weather events (Dilley et al., 2005). Thus, any successful adaptations should be able to cope with the short-term climate variability as well as reduce the negative impacts of climate change in the long term (Saba et al., 2013; Lobell, 2014). Hertel and Lobell (2014) distinguished between three categories of adaptation: (i) adaptation options based on current technology which can also identified as autonomous adaptation, (ii) adaptation involving a new technologies, and (iii) adaptations involving the institutional environment within which the producer is operating such as markets and policy and resulting from planned adaptation. Adjustments in planting and harvesting dates, varieties of crops to be grown (including combination between crops and cultivars as intercrop or the use of existing varieties more resistant to climate-induced stress), increase planting density and/or fertilizers use, use of crop residue as mulch are examples of options already available to farmers in West Africa to adapt to climate variability and change. Breeding more resilient crop varieties (Rötter et al., 2015), advanced breeding methods including more effective root system size, dehydrin genes, phenotyping (Araus et al., 2012; Setter, 2012; Vadez et al., 2012; Amelework et al., 2015); innovating water harvesting techniques (Lebel et al., 2015; Rockström and Falkenmark, 2015) belong to the second category of adaptation options. In the third category defined by Hertel and Lobell (2014), fertilizer subsidies, crop insurances (Berg et al., 2009), credits, climate services (access and use of weather and seasonal forecasts; Sultan et al., 2010; Roudier et al., 2012, 2014, 2016) are such important changes in the institutional and market environment of West Africa that would affect producer decisions. Assessing various possible adaptation options and their uncertainties is crucial for optimal prioritization of adaptation investments for supporting adaptation strategies in West Africa that may counteract the adverse effects of climate change. However, pointing out the most promising adaptation options remains challenging since there is a large scatter of possible results across locations and situations, indicating the need for a more contextual approach on regional and local scales (Challinor et al., 2014). We will thus give some examples of some recent studies who quantified the potential of adaptation for major crops in West Africa showing sometimes apparent contradictory and crop-specific results.

#### Millet and sorghum

These two crops are among the main staple crops of sub-Saharan West Africa (64% of the total cereal production in 2000; FAOSTAT, 2012 data). On-farm surveys have shown the dominance of traditional cultivars of sorghum and millet characterized by a strong sensitivity to photoperiod (Traore et al., 2011). Photoperiod sensitivity would likely present some advantages in the event of future change in the timing of the rainy season. Indeed, it allows for flowering at the end of the rainy season for a wide range of planting dates and avoids incomplete grain filling, a problem for late maturing varieties faced with water shortage at the end of the rainy season (Dingkuhn et al., 2006). Furthermore, Sultan et al. (2013) found that traditional photoperiod-sensitive cultivars are less affected by temperature increase since the photoperiod limits the reduction of the crop duration. On the opposite, adverse impacts of climate change have been found to be the lowest on mean yield and yield variability for photoperiod-insensitive cultivars, as their short and nearly fixed growth cycle appears to be more resilient to the seasonality shift of the monsoon, thus suggesting shorter season varieties could be considered a potential adaptation to ongoing climate changes (Sultan et al., 2014). This result is consistent with the study from Kouressy et al. (2008), which demonstrated that potentially high-yielding and photoperiod-insensitive cultivars display an advantage where the rainy season is short. Modeling studies (Turner and Rao, 2013; Sultan et al., 2014) suggest that while increasing fertilizer inputs and restoring nutrients imbalance in low-input, smallholder, sorghum farmers of Africa would increase overall food production and have fundamental benefits increasing food security (Vitousek et al., 2009), the trade-off is that it would increase the sensitivity of those systems to climate variability and increase adverse impacts of climate change.

Several studies also investigated new technologies for mitigating the adverse impacts of climate change on millet and sorghum production. Adiku et al. (2015) used two crop models DSSAT and APSIM to simulate millet cultivars adapted to future climate conditions. They found positive effects on crop yield whereas the benefits depend on the location, the crop and the climate model used for the simulation. Sultan et al. (2013) also found advantages of breeding varieties with higher thermal requirements which can partly counteract the shortening of crop-cycle duration in a warmer climate. Guan et al. (in press) used two crop models APSIM and SARRA-H to assess five possible and realistic adaptation options for the production of sorghum (late sowing, increase planting density and fertilizer use, increasing cultivars' thermal time requirement, water harvesting, and increase resilience to heat stress during the flowering period). They found that most proposed adaptation options are not more beneficial in the future than in the historical climate so that they do not really reduce the climate change impacts. Increased temperature resilience during grain number formation period is the main adaptation that emerges from this study.

#### Maize

Maize is the most important staple food and accounts for nearly 20% of total calorie intake in sub-Saharan Africa (SSA) (FAOSTAT, 2012, data). In their meta-analysis, Challinor et al. (2014) compared the effect of climate change on maize yields in the Tropics with and without adaptation; adaptation options including changes in planting dates, fertilizer use, irrigation, cultivar or other agronomic options. They concluded that in contrast to what has been published for wheat and rice in the temperate latitudes, there is no effect of adaptation in the Tropics and little evidence for the potential to avoid yield loss in maize yield since the varieties of crop grown are already adapted to high temperatures. Similar results were also found by Deryng et al. (2011) who reported substantial yield losses in developing countries located in the Tropics for maize even after allowing for adjustment of planting dates and varieties grown. Using simulations from the GEPIC model in Sub-Saharan Africa, Folberth et al. (2014) investigated different intensification options for growing maize under climate change. They found that intensive cultivation is predicted to result in lower yields under future climate conditions and increased soil erosion while eco-intensification shows better yields. However, yield losses are simulated in all management scenarios toward the end of the century suggesting a limited effect of eco-intensification as a sole means of adapting agriculture to climate change. Finally, promising results of rainfall harvesting have been found by Lebel et al. (2015) which found that applying this technique to maize cultivation across Africa could mitigate 31% of yield losses attributable to water stress and increase maize yields by 14–50% on average under the projected climatic conditions of the 2050s.

#### Groundnut and yam

Groundnut is an important crop for Nigeria, southern Mali, Ivory Coast, Burkina Faso, Ghana, and Senegal. Parkes et al. (2015) investigated the benefits of breeding cultivars of groundnuts with heat and water stress resistance as well as the potential of marine cloud brightening to reduce the rate of crop failures in West Africa using the GLAM model. The authors found that climate change will increase mean yields of groundnut and reduce the risk of crop failure in West Africa. This projected increase in yields is due to the carbon dioxide fertilization effect also to increased seasonal rainfall in the unique GCM simulation used in this study. Parkes et al. (2015) investigated the benefits of breeding cultivars of groundnuts with heat and water stress resistance as well as the potential of marine cloud brightening to reduce the rate of crop failures in West Africa. They found that water stress, rather than heat stress, is the main cause of crop failure in current and future climate and also demonstrated a positive impact of marine cloud brightening.

Yam is the second most important crop in Africa in terms of production after cassava. Srivastava et al. (2015) simulated the advantages of specific adaptation strategies using the EPIC model. They found that changing solely sowing date may less effective in reducing adverse climatic effects than adopting late maturing cultivars. Yet, combining different options such as coupling irrigation and fertilizer application with late maturing cultivars, highest increase in the yields could be realized.

#### Cassava

Using the EcoCrop model to investigate the response of important staple food crops for Africa including maize, millets, sorghum, banana, and beans to climate projections by 2030, Jarvis et al. (2012) found that cassava reacted very well to the predicted future climate conditions compared to other crops. Whilst most simulated crops in Africa were predicted to experience decreases in overall suitability in Africa, cassava always outperformed or (in the worst case) equaled the average and appeared as a highly resilient staple crop. Crop improvements toward greater drought tolerance and heat tolerance in localized pockets of West Africa and the Sahel could bring some additional benefits.

## Summary and conclusions

In this paper, an extensive review of the recent literature on the West African climate and impacts is used to draw a general picture of the main features of the regional climate, the associated observed variability, the future change as well as expected impacts and potential for adaptation in the agriculture sector.

The dominant role of the WAM in determining the regional climate is highlighted, and the importance of the global SST in driving the multi-time scales variability is described (Rodríguez-Fonseca et al., 2015). In particular, the relationship of the WAM precipitation variability with the tropical ocean SST at the interannual time scales (Rowell, 2001; Polo et al., 2008; Losada et al., 2010; Mohino et al., 2011b), and with the extratropical ocean SST at multidecadal time scales (Zhang and Delworth, 2006; Ting et al., 2009; Mohino et al., 2011a; Villamayor and Mohino, 2015), is illustrated. The long lasting wet phase characterizing the Sahelian precipitation in the twentieth century up to the 70s, and the following severe drought affecting the Sahel culminating in the 80s, have been related principally to the SST variability associated with the Atlantic multidecadal variability (Mohino et al., 2011a). At the turn of the twenty-first century, the Sahel experienced a slight recovery of precipitation (Panthou et al., 2014; Maidment et al., 2015; Sanogo et al., 2015), but the attribution of this recovery is still debated. On the one hand, it is attributed to the differential warming between extratropical and tropical SST in the Northern Hemisphere, favoring the northward displacement of the ITCZ (Park et al., 2014). On the other hand, the recovery is attributed to the regional radiative warming produced by the CO_2_ direct forcing, inducing a thermodynamic feedback on the monsoon system (Dong and Sutton, 2015). The rainfall recovery has been characterized by a modification of the precipitation regime, with higher intensity rainfall events concentrated in less rainy days (Panthou et al., 2014). Moreover, a widespread warming of the North African subcontinent, and an increase in the occurrence of climate extremes, such as heat waves ad hot summers, has been observed (Fontaine et al., 2013; Moron et al., 2016).

The same tendencies in temperature, precipitation and climate extremes are projected in the twenty-first century, in all the moderate-to-high emission scenarios, with the amplitude of the climate change signal growing proportionally with the projected global warming. The intensification of the hydrological cycle in the recent decades and in future projections has also been detected in in the world's dry and wet regions, leading to an increased risk of flooding in dry regions as the climate warms (Donat et al., 2016). However, the future projections of the West African climate are affected by large uncertainties, especially regarding the monsoonal precipitation. Indeed, although around 50% of the CMIP5 GCMs agrees on the future positive trend, around 25% of the models project the opposite situation, weakening the prevision (Biasutti, 2013). The origin of this uncertainties is two-fold. On the one hand, the biases characterizing the SST simulated by the atmosphere-ocean climate models, which affect the mechanisms driving the multidecadal variability of the WAM system (Roehrig et al., 2013; Rowell, 2013). On the other hand, the diverse sensitivity of climate models to the effect of the projected increase in CO_2_ concentration, which induces wet anomalies through the direct radiative warming of the surface at the regional scale, but at the same time inhibits the precipitation when the radiative forcing is mediated by the global SST warming (Bony et al., 2013; Gaetani et al., 2016). Climate modeling of West Africa at the regional scale shows promising improvements of the GCM performances, although large uncertainties still persist. Firstly, RCMs are inevitably affected by the biases of the driving GCMs (Dosio et al., 2015). Secondly, RCMs experiments show high sensitivity to the physical parametrization, especially regarding convection (Klutse et al., 2016), which is crucial for the simulation of the monsoonal rainfall. Therefore, the climate modeling community is pushed for a further effort to improve the modeling of West African climate, in the direction of both understanding the physical mechanisms and reducing the climate model shortcomings.

There are many complex processes that drive the response of crop yield to such climate changes. These processes can act in a competing way as we can expect from the role of increased atmospheric CO_2_ concentration which increase crop yield while warmer mean temperatures are likely to lead to crop yield losses. Such processes can interact together and their importance might depend on the region, the scale and the crop. The complexity of the risk posed by climate change and possible adaptation strategies have called for a number of climate change assessment studies especially in Africa where this risk can severely affect food security and impede development. Despite a large uncertainty in the published results and diverging future projections of summer monsoon rainfall which is key for rain-fed agriculture, a robust evidence of yield loss in West Africa emerges from these studies. This yield loss is mainly driven by increased mean temperature while potential wetter conditions as predicted in Central Sahel or elevated CO_2_ concentrations for C3 crops and C4 crops in the arid zones of the Sahel can partly or totally counteract this effect. On the opposite, yield losses will be the highest for C4 crops in the Soudano-Sahelian zones and in areas where rainfall is expected to decrease like in the Western part of the Sahel. Identifying the most promising adaptation options is even more uncertain since uncertainty about climate impacts is then cumulated with uncertainty about the effectiveness of adaptations. Most adaptation options illustrated in this review are implemented in process-based crop models to adjust cropping systems (change in varieties, sowing dates and density, irrigation, fertilizer management) to future climate. Results of the cited studies are crop and region specific and no clear conclusions can be made regarding the most effective adaptation options.

Although substantial progress has been made in the assessment of the effect of climate change on crop yield and potential for adaptation in West Africa, large gaps still exist. Important processes like the effect of heat stress or ozone are missing in crop models (Ewert et al., 2015), most effort on model development and intercomparison are biased toward major crops in temperate regions and the African region generally suffers from a lack of sufficient data for accurate calibration and validation of crop models (Lobell and Burke, 2010). Furthermore, specific crop management options and cultivars of low intensive systems as mainly found in West Africa (mulching, species mixtures, intercropping and reduced tillage technologies) are not well represented in crop models (Hertel and Lobell, 2014; Ewert et al., 2015). If recent progress has been made to quantify the potential for adaptation in integrated assessment and modeling approaches linking biophysical and economic models (Patt et al., 2010; Ewert et al., 2015), these approaches are built on assumptions which are more appropriate for the high income and developed countries with high adaptive capacity. Hertel and Lobell (2014) concludes that they present a risk to underestimate the impacts of climate change in the Tropics and a risk of overstating the efficiency of adaptations in regions like Sub-Saharan Africa.

As suggested by Challinor et al. (2009), an objective quantification of impacts uncertainty is a necessary step to go beyond syntheses or meta-analyses of published studies with large heterogeneity resulting from inherently uncoordinated studies. Large ensemble of climate simulations, downscaling techniques and crop simulation ensembles including different modeling approaches and sensitivity analyses are necessary for improved understanding of how climate uncertainties and errors propagate into impact estimates, a better quantification of crop model uncertainty as well as a better quantification of downscaling and bias-correction uncertainty (Ramirez-Villegas et al., 2013). In this respect, coordinated efforts such as the AgMIP initiative which aims to improve agricultural models including biophysical and socio-economic approaches at various scales and develop common protocols to systematize modeling for the assessment of climate change impacts on crop production represents a promising way toward more robust results (Rötter, 2014). While they are crucially lacking in Sub-Saharan Africa, observations are also a key to go forward in the quantification of uncertainty and possible reduction of its range. Most modeling work on climate impacts assessment needs quality data to validate and bias-correct climate simulations, calibrate, validate and force crop models or evaluate cropping systems adaptation. Improvement of quality, accessibility of data (including weather, soil, on-farm and experimental crop data, socio-economic data) as well as support for maintaining data over time and collecting long-term time series is of high importance in Sub-Saharan Africa. Finally, if there is evidence that farmers and farming systems are highly resilient to environmental changes, adaptation to climate change needs to be supported and facilitated by governmental, institutional and macro-economic conditions (Challinor et al., 2007). Adaptation to climate change cannot be achieved without a considerable institutional and political commitments for technical support or access to credit for instance (Thornton et al., 2011) and many of institutional, economic, informational, and social constraints are still ignored in modeling approaches of adaptation (Hertel and Lobell, 2014) which need to better account for both the biophysical and socio-economic determinants and specificities of agricultural systems in Africa.

## Author contributions

All authors listed have made substantial, direct and intellectual contribution to the work, and approved it for publication.

### Conflict of interest statement

The authors declare that the research was conducted in the absence of any commercial or financial relationships that could be construed as a potential conflict of interest.
